# Paraceutesis of the Pericardium

**Published:** 1880-02

**Authors:** 


PARACEUTESIS OF THE PERICARDIUM. A Consideration of the Surgical Treatment of Pericardial Effusions. By John B. Roberts, A.M., M.D., Lecturer on Anatomy in Philadelphia School of Anatomy, etc., etc., with illustrations. J. B. LIPPINCOTT & Co: Philadelphia. 1880.

